# When is a forest a forest? Forest concepts and definitions in the era of forest and landscape restoration

**DOI:** 10.1007/s13280-016-0772-y

**Published:** 2016-03-09

**Authors:** Robin L. Chazdon, Pedro H. S. Brancalion, Lars Laestadius, Aoife Bennett-Curry, Kathleen Buckingham, Chetan Kumar, Julian Moll-Rocek, Ima Célia Guimarães Vieira, Sarah Jane Wilson

**Affiliations:** 1 University of Connecticut, 75 North Eagleville Road, Unit 3043, Storrs, CT 06269-3042 USA; 2 Universidade de São Paulo/ESALQ, Av. Pádua Dias 11, Piracicaba, SP 13418-900 Brazil; 3 World Resources Institute, 10G Street NE, Washington, DC 20002 USA; 4 School of Geography and the Environment, Environmental Change Institute, University of Oxford, South Parks Road, Oxford, OX1 3QY UK; 5 CIFOR, Bogor, Indonesia; 6 International Union for the Conservation of Nature, 1630 Connecticut Ave. N.W., Washington, DC 20009 USA; 7 Museu Paraense Emilio Goeldi, CP 399, Belém, PA CEP 66060-310 Brazil; 8 IFRI, University of Michigan, 440 Church Street, Ann Arbor, MI 48105 USA; 9 11407 Symphony Woods Lane, Silver Spring, MD 20901 USA; 10 3298 Greenwood Heights Drive, Kneeland, CA 95549 USA

**Keywords:** Deforestation, Forest assessment, Forest management, Landscape, Plantation, Reforestation, Restoration

## Abstract

We present a historical overview of forest concepts and definitions, linking these changes with distinct perspectives and management objectives. Policies dealing with a broad range of forest issues are often based on definitions created for the purpose of assessing global forest stocks, which do not distinguish between natural and planted forests or reforests, and which have not proved useful in assessing national and global rates of forest regrowth and restoration. Implementing and monitoring forest and landscape restoration requires additional approaches to defining and assessing forests that reveal the qualities and trajectories of forest patches in a spatially and temporally dynamic landscape matrix. New technologies and participatory assessment of forest states and trajectories offer the potential to operationalize such definitions. Purpose-built and contextualized definitions are needed to support policies that successfully protect, sustain, and regrow forests at national and global scales. We provide a framework to illustrate how different management objectives drive the relative importance of different aspects of forest state, dynamics, and landscape context.

**Electronic supplementary material:**

The online version of this article (doi:10.1007/s13280-016-0772-y) contains supplementary material, which is available to authorized users.

## Introduction

We live in an era of unprecedented environmental change, motivating equally unprecedented global actions to protect and restore forest ecosystems (Aronson and Alexander 2013). These efforts could fail to achieve their ambitious goals if they are not informed by clear and appropriate concepts and definitions of forests. Forest definitions provide the conceptual, institutional, legal, and operational basis for the policies and monitoring systems that drive or enable deforestation, forest degradation, reforestation, and forest restoration (van Noordwijk and Minang 2009).

Forest concepts and definitions influence how we assess and interpret forest transitions—the change over time in the balance between forest loss and forest gain within a geographic region—where both loss and gain are defined in terms of tree canopy cover. Forest gain is not the mirror-image opposite of forest loss. In most cases, forest loss is concentrated and abrupt, and can be clearly documented with a sequence of satellite imagery or aerial photos. Forest gain, in contrast, is a highly variable, dispersed, and protracted process that is challenging to document and monitor with commonly used forest definitions and technology (Chazdon 2014). The functional, structural, and compositional properties of new tree cover differ substantially from those of the forest or non-forest ecosystems they replace (Brown and Zarin 2013; Tropek et al. 2014). New tree cover can take many forms, from spontaneous natural regeneration to single-species plantations of non-native trees. Local forest disturbance and ingrowth that accompany tree harvesting and silvicultural management are also challenging to detect and monitor. Differentiating among these different forms of tree cover gain poses a far greater challenge than identifying areas where forest cover has been removed. Widely used forest definitions that perform well for assessing rates of deforestation—as measured by rates of transformation of forest to non-forest land uses—have not proved useful in assessing forest restoration and regeneration.

**Fig. 1:**
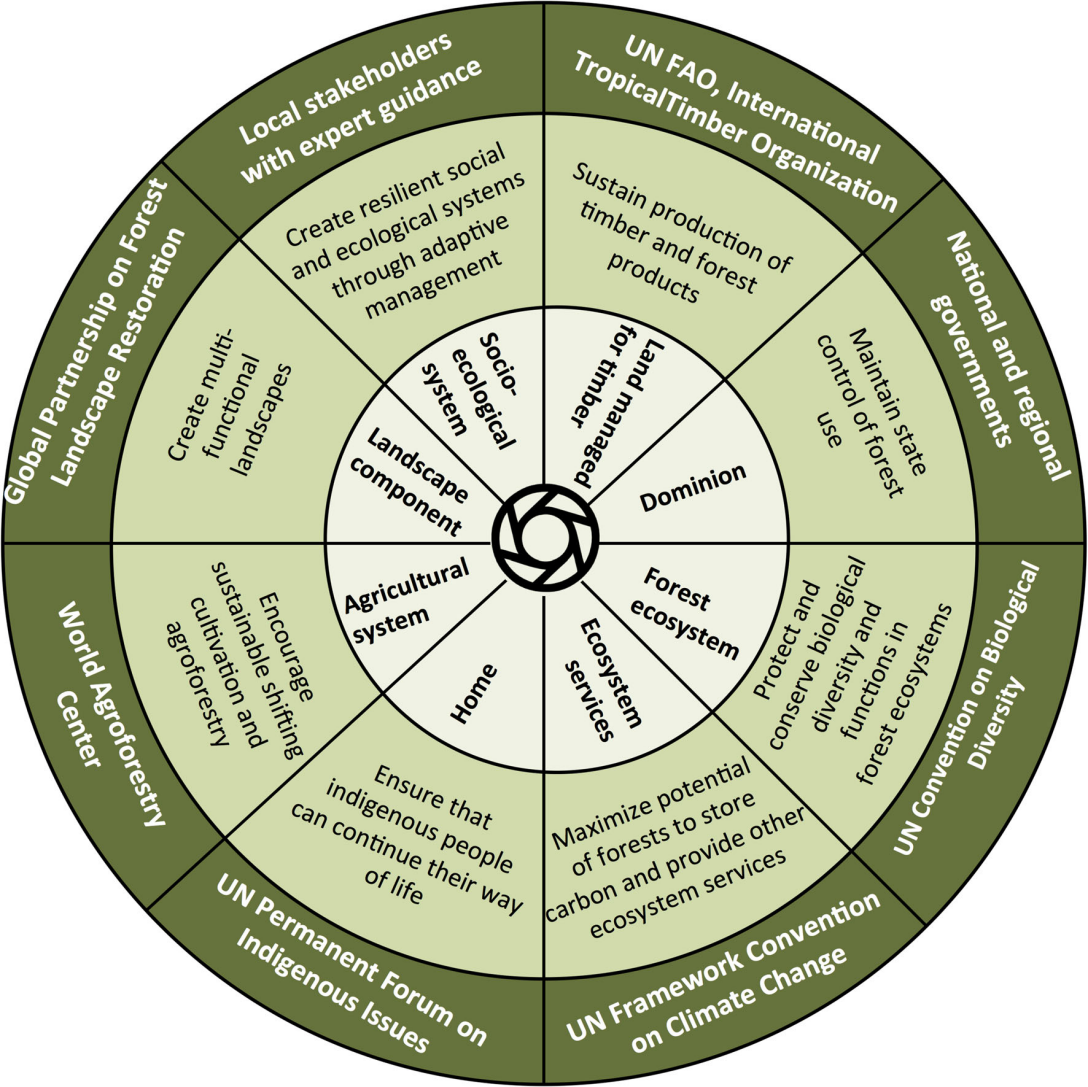
Different management objectives form the basis from which a forest is conceptualized and definitions are created. The *inner circle* shows how a forest can be viewed through different lenses, emanating from the different management objectives shown in the *middle circle*. Each objective provides a perspective from which specific definitions are created. The *outermost circle* describes institutions whose mission is associated with each management objective and forest definition

Forests are viewed, defined, assessed, and valued through different lenses. From different vantage points, forests can be seen as a source of timber products, an ecosystem composed of trees along with myriad forms of biological diversity, a home for indigenous people, a repository for carbon storage, a source of multiple ecosystem services, and as social-ecological systems, or as all of the above (Fig. 1). In addition, a fundamental and commonly misunderstood distinction exists between the actual features of land and its legal designation. From the “land cover” perspective, forests are viewed as ecosystems or vegetation types supporting unique assemblages of plants and animals. But from the “land use” perspective, forests are landholdings that are legally designated as forest, regardless of their current vegetation. Within this construct, a legally designated “forest” can actually be devoid of trees, at least temporarily. No single operational forest definition can, or should, embody all of these dimensions.

The world is entering a new era of ecosystem restoration motivated by the Aichi Targets; the Bonn Challenge to restore 150 million hectares of degraded and deforested land by 2020; and the New York Declaration on Forests, launched at the UN Climate Summit 2014. Article 5 of the Paris Agreement produced by the 2015 UN Climate Change Conference places forest conservation, enhancement, and sustainable management in the forefront of climate mitigation policies. To meet ambitious global restoration targets, policy makers, governments, scientists, and agencies need to adopt a richer concept of a forest than the dominant FAO definition that has governed forest policy to date (Box 1). A diverse set of forest definitions is needed to capture this forest concept in all its dimensions.

In this Perspective, we propose that forest definitions be applied more carefully and deliberately to achieve specific management objectives, rethinking how new forms of tree cover are classified and evaluated within different management and policy contexts. First, we present a historical overview of forest concepts and definitions and link them with distinct perspectives and objectives for forest use. We discuss forest concepts and frameworks that have motivated different forest definitions globally over the past three centuries, noting that commonly used definitions created to measure changes in forest stocks have limited utility for assessing and monitoring new and diverse forms of forest cover, which we refer to as “reforests.” “Reforests” collectively constitute forest gain, and are increasing dramatically in global importance (Chazdon 2014). We then illustrate how the use of a particular forest definition can influence policy-making, monitoring, and reporting regarding forests, through documented case studies. We emphasize the need to distinguish different types of “reforests” based on their origins, dynamic properties, and landscape settings. Building on these fundamental properties of forest types, we present a framework to illustrate how definitions applied to specific purposes vary in the importance of seven criteria: (1) value for timber; (2) value for carbon storage; (3) improving livelihoods of forest-dependent people (4) whether forests are natural or planted; (5) whether forests are pre-existing or newly established; (6) whether forest are continuous or fragmented; and (7) whether forests are composed of native or non-native species (Table 1). We conclude with a call for a more nuanced and diversified approach to defining forests and “reforests” that can distinguish natural from planted forests and forests damaged by logging from second-growth forests, and can be used to track the dynamics of regrowing forest patches within agricultural landscapes.

**Table 1:** A preliminary framework of criteria for forest definitions that vary in importance for specific forest management objectives. The framework focuses on ecological and production criteria, but it is also important to include social and cultural criteria for defining and assessing forests. Criteria for definitions are not static, as forest management objectives will need to adapt to changing circumstances imposed by climate change, government policies, or international markets

Criteria for definition	Forest management objective			
	Conservation of natural ecosystem	Timber management	Increase carbon stocks	Landscape restoration
Key properties for forest definition	Ecological properties, native biodiversity, and dominance of native trees	Legal designation, areal extent, size and density of trees	Areal extent, size and density of trees, land use history	Uses of trees, multiple ecosystem services, livelihoods, biodiversity conservation status
Value for timber production	Not important	Very important, as main objective of management	Important in terms of value for carbon stocks	Important for local livelihoods and smallholders
Value for carbon storage	Important for ecosystem functioning and climate mitigation	Important for management and climate mitigation	Very important as main objective	Important for ecosystem functioning and climate mitigation
Livelihoods of forest-dependent people	Important in the context of indigenous/community reserves	Important only within forestry sector	Not important	Very important as they are major stakeholders
Distinction between planted and natural forest	Very important, because of ecological properties and native biodiversity	Important, because of differences in tree properties and sensitivity in some markets	Not important, because the origin of carbon stock does not matter	Important, because of differential cost and benefits, effects on multiple ecosystem services, and forest-based livelihoods
Distinction between pre-existing and newly established forests (reforests)	Very important because successional stages vary in ecological properties and native biodiversity	Important because of forest management, tree properties and timber yield	Very important because of differences in carbon stocks and additionality constraints	Very important because of different ecological and economic properties and additionality
Distinction between continuous and fragmented forest	Very important because of impacts on ecological properties, connectivity and biodiversity conservation	Important because of sensitivity in some markets to origins of timber sources	Not important because the origin of carbon stock does not matter	Very important because of effects on ecosystem services, connectivity, and biodiversity conservation
Distinction between native and non-native trees in forest	Very important because of impacts on ecological properties and native biodiversity	Important because of differences in tree and wood properties	Not important because the origin of carbon stock does not matter	Important because of effects on ecosystem services and biodiversity conservation

## Forest definitions reflect forest management objectives

**Fig. 2:**
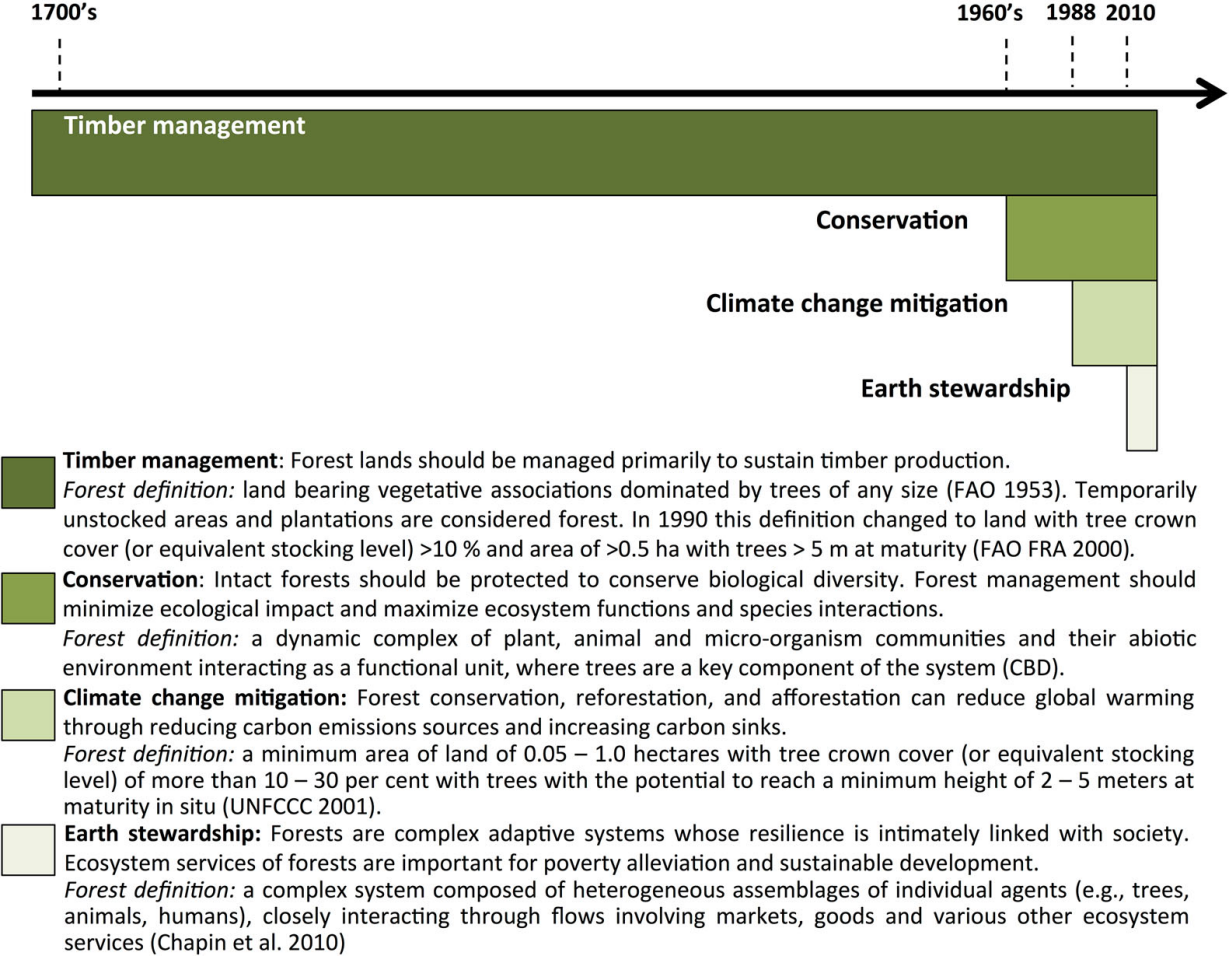
Forest definitions emerge from prevailing objectives of use and management. Since the mid-twentieth century, forest management objectives and definitions have diversified, with new ones being added to earlier more entrenched and legitimized ones. Similarly, forest management policies have broadened their objectives, focusing not only on sustainable timber production, but gradually incorporating non-timber forest products, biodiversity conservation values, ecosystem services delivery, human well-being, landscape approaches, adaptive management, and socio-ecological resilience

Approaches to forest management are embedded within political ecology. As forest management objectives respond to changing societal needs and values, so should definitions. Over time, new management objectives have been added to preexisting ones in a cumulative process (Fig. 2; Supplementary Material S1). Although people have been managing forests for millennia for diverse uses, we begin our historical overview in the 1700s in Germany, as this period marked the development of theory-based forest management to sustain a high timber yield, and the concept of forests as timber (Schmithüsen 2013). Earlier historical concepts and definitions of forest are discussed by Putz and Redford (2010). This objective required that forest be defined for the purpose of managing yield-related characteristics over large spaces (many stands of timber) and long time periods (more than one rotation) in order to assess the amount of wood that could be harvested (Puettmann et al. 2009). Within this historical and geographical context, the distinction between natural and planted forest was not important.

Concern about shortages in forest products following World War II motivated the United Nations Food and Agricultural Organization (FAO) to conduct the world’s first global forest inventory (Holmgren and Persson 2002). In 1948, the FAO adopted a forest definition suitable for assessing wood harvesting potential (Box 1). FAO’s definition, agreed on by all its members, is the first to be used by all countries for harmonized reporting; the definition adopted by FAO remains the most widely used forest definition today (Grainger 2008).

**Box 1:** Forest definitions adopted by major international environmental and forestry organizations

*United Nations Food and Agriculture Organization (FAO; 2000)* Land with tree crown cover (or equivalent stocking level) of more than 10 % and area of more than 0.5 ha. The trees should be able to reach a minimum height of 5 m at maturity in situ. May consist either of closed forest formations where trees of various storeys and undergrowth cover a high proportion of the ground; or open forest formations with a continuous vegetation cover in which tree crown cover exceeds 10 %. Young natural stands and all plantations established for forestry purposes which have yet to reach a crown density of 10 % or tree height of 5 m are included under forest, as are areas normally forming part of the forest area which are temporarily unstocked as a result of human intervention or natural causes but which are expected to revert to forest
*United Nations Framework Convention on Climate Change (UNFCCC; 2002)* A minimum area of land of 0.05–1.0 ha with tree crown cover (or equivalent stocking level) of more than 10–30 % with trees with the potential to reach a minimum height of 2–5 m at maturity in situ. A forest may consist either of closed forest formations where trees of various storeys and undergrowth cover a high proportion of the ground or open forest. Young natural stands and all plantations which have yet to reach a crown cover of 10–30 % or tree height of 2–5 m are included under forest, as are areas normally forming part of the forest area which are temporarily unstocked as a result of human intervention such as harvesting or natural causes but which are expected to revert to forest
*United Nations Convention on Biological Diversity (UN*-*CBD; 2010)* A land area of more than 0.5 ha, with a tree canopy cover of more than 10 %, which is not primarily under agriculture or other specific non-forest land use. In the case of young forest or regions where tree growth is climatically suppressed, the trees should be capable of reaching a height of 5 m in situ, and of meeting the canopy cover requirement
*United Nations Convention to Combat Desertification (UN*-*CCD; 2000)* Dense canopy with multi-layered structure including large trees in the upper story;
*International Union of Forest Research Organizations (IUFRO; 2002)* A land area with a minimum 10 % tree crown coverage (or equivalent stocking level), or formerly having such tree cover and that is being naturally or artificially regenerated or that is being afforested

Environmental movements arising in the 1960s generated new forest management objectives based on the ecological concept of forest as natural ecosystems (Figs. 1, 2), mobilizing individuals and newly formed national and international organizations to conserve nature and halt habitat loss, environmental degradation, and biodiversity decline (Supplementary Material S1). These organizations used forest definitions emphasizing forest ecosystems features, and their distribution across terrestrial ecoregions. Over time, conservation became increasingly incorporated into forest management objectives, as evidenced internationally by the creation of the Convention on Biological Diversity and the adoption of the Forest Principles (1992), the Forest Stewardship Council (1993), and comprehensive regional monitoring and reporting frameworks including the Helsinki Process (initiated in 1990) and the Montreal Process (1994; Supplementary Material S1).

In the 1980s, concerns about climate change led to the establishment of the Intergovernmental Panel on Climate Change (1988) and the creation of the UN Framework Convention on Climate Change (1992), initiating a new forest management objective: forests as carbon stocks (Figs. 1, 2). The Kyoto Protocol contains the terms reforestation and afforestation which subsequently had to be defined and operationalized in this context (Box 1). The adoption of the Bali Action Plan in 2007 gave rise to the Forest Carbon Partnership Facility and the UN-REDD Programme. Biomass and carbon density became the metrics of forest monitoring and assessment (Saatchi et al. 2011). Attempts to quantify and monetize carbon sequestration and other ecosystem services were expanded to incentivize forest protection and reforestation through payments for ecosystem services (Wunder 2007), and the creation of the Intergovernmental Panel on Biodiversity and Ecosystem Services (2012) formally expanded this perspective of forests as providers of multiple ecosystem services linked to their biodiversity.

We are on the cusp of a new perspective of forests (and other ecosystems) based on the concepts of resilience, earth stewardship, and integrated landscape planning (Fig. 2; Chapin et al. 2011; Sayer et al. 2013). Forests and their surrounding landscapes are viewed as complex adaptive systems, whose properties arise through self-organization and interactions among internal and external components, including human societies (Messier et al. 2015). A key component of this integrated approach is managing forests at the landscape level, which requires balancing multiple types of ecosystems with the needs of multiple sets of actors who use them. Forests are not defined as isolated entities, but as integral components of dynamic, multi-functional landscapes. In contrast to the forest concepts previously discussed, the landscape approach requires a broader concept of forest that blurs the boundaries of definitions applied by existing forestry, agriculture, and conservation institutions.

Multiple concepts and definitions of forest now coexist, as they should. Yet, aligning their objectives and roles in policy-making and governance remains a major challenge. More than ever we need clear forest definitions that are applied to achieve specific objectives for managing forests and reforests in the world’s rapidly changing landscapes. Perverse and unintended consequences can and do arise when definitions and inventory methods developed to demarcate and assess timber stock and growth are used beyond their scope of useful relevance, e.g., for making policy relating to biodiversity, ecosystem services, and non-timber forest products.

## Forest definitions and policy

Forest definitions shape environmental policies in multiple ways at global, national, and regional scales. The conceptual frameworks that emerge from contemporary social and political movements influence the policies and decisions that ultimately determine the fate of forests and the people near and far that rely on them for sustenance, services, and products. But forest definitions are also constrained by feasibility considerations emanating from available data collection technology, human capacity, and budgetary allocations, as well as by purpose. Definitions used for surveying the status and change in forest growing stock at national scale, for example, tend to contain thresholds determined by technically conditioned cost-benefit considerations, such as a minimum patch size (e.g., 0.5 ha) and a minimum tree size (e.g., 5 cm diameter at breast height or 5 m height, a threshold that is more relevant to ground-based inventories than remote sensing surveys).

FAO’s Forest Resources Assessment (FRA) defines forest as *land* with certain characteristics that determine its demarcation (Box 1). Under this definition, harvesting or clearing of all trees from a tract of land does not constitute deforestation in cases “where the forest is expected to regenerate naturally or with the aid of silvicultural measures within the long-term” (FAO 2001, p. 25). “Deforestation” requires a change in land use from forest to non-forest, consistent with the objective of tracking and maintaining land to be used for timber production. The FRA definition is not appropriate for assessing and monitoring forest degradation (Sasaki and Putz 2009; Putz and Redford 2010). For example, forests in Tanzania would remain classified as forests with no measurable deforestation even if 88 % of the trees were removed and up to 87 % of forest carbon was lost (van Noordwijk et al. 2009). Moreover, new forests, including restored forests and early stages of spontaneous natural regeneration, go unnoticed if they fail to satisfy the FAO definition.

Forest definitions have a similar effect on approaches to afforestation, defined by FRA as “establishment of forest through planting and/or deliberate seeding on land that, until then, was not classified as forest” (FAO 2010, p. 13). The consequences of applying this forest definition extend beyond forest ecosystems. Tree plantations on lands that lie within natural grassland biomes are considered forests by the FRA definition, although they are also distinguished by FAO as planted forests (FAO 2004). The FRA forest definition does not distinguish tropical dry forests from mesic savannas, which differ in qualitative rather than structural aspects of the vegetation. If planted or naturally regenerating trees can grow in savannas under conditions of fire suppression, then the FRA definition will consider the tree-covered portions of the savanna as being forest.

Use of different definitions leads to vastly different estimates of national and global forest cover (Grainger 2008) and observed rates of forest gain and loss (Keenan et al. 2015; Box 1). For example, the estimate of global forest area increased by 300 million ha (approximately 10 %) between 1990 and 2000 simply because the FRA changed its global definition of forest, reducing the minimum height from 7 to 5 m, reducing the minimum area from 1.0 to 0.5 hectares (ha) and reducing minimum crown cover from 20 to 10 % (FAO 2000). In Australia, where trees often occur in open vegetation formations, this reclassification led to the acquisition of an additional 118 million ha of forest (Matthews 2001).

In many cases, forest assessments do not distinguish between land covered by natural and planted forests (Sasaki and Putz 2009). Thus, if natural forests are cleared and replaced with plantations, no net loss of forest cover is reported (Brown and Zarin 2013). Furthermore, tree harvesting from managed plantations is not distinguished from clearance from natural forest (Petersen et al. 2016). High rates of natural forest conversion have persisted in some tropical countries, in part because their operational forest definitions do not distinguish between monoculture plantations and natural forests (Zhai et al. 2014; Box 1). Using widely adopted structural forest definitions based solely on tree height, minimum area, and crown cover (Box 1) without complementary analysis based on additional definitions, countries can show zero net deforestation or even a gain in forest extent, even while having converted considerable areas of natural forest within the same time interval (Tropek et al. 2014). In mapping global tree cover, Hansen et al. (2014) included plantations of oil palm, rubber, and tree monocultures in their definition of forest cover. The definition used for the 2015 Forest Resources Assessment (FRA) excludes fruit tree plantations, oil palm plantations, olive orchards, and agroforestry systems with crops grown under tree cover, but includes rubber, cork oak, and Christmas tree plantations (FAO 2012). According to the FRA, replacing a rubber plantation with an oil palm plantation results in a loss of both forest cover and forest plantation area (Keenan et al. 2015). Because bamboo stands meet the structural criteria for forest defined by FRA, bamboo harvesting and trade must adhere to many standards developed for timber (Buckingham et al. 2013). Inconsistently applied definitions also lead to unclear forest policy: the government of Peru does not necessarily define plantations as forests, but oil palm is considered a highly suitable tree species for “reforestation” in degraded areas (Bennett-Curry, personal communication).

Although economic forces are the proximate drivers of deforestation (Geist and Lambin 2002), defining tree plantations as forests can compromise the quality of information available to support and enforce protection and governance of natural forests at national and subnational scales. From 1988 to 2005, while the area of natural forests of Hainan Island, China decreased by 22 %, the area of rubber and pulp plantations increased more than 400 % and the total forest cover remained unchanged (Zhai et al. 2012). Rubber plantations have replaced nearly all of the natural forest in Xishuangbanna, China (Li et al. 2007). Similar trends in replacement of native old-growth and second-growth forest by exotic tree plantations have been documented in southern Chile (Zamorano-Elgueta et al. 2015), Thailand (Leblond and Pham 2014), and India (Puyravaud et al. 2010). Across SE Asia, nearly 2500 km^2^ of land previously classified as natural vegetation with tree cover was converted to rubber plantations between 2005 and 2010 (Ahrends et al. 2015).

Depending on the policy environment, forest definitions can have major consequences for the fate of small forest fragments and areas with sparse tree cover, which constitute substantial amounts of the remaining areas of natural forest in many regions. Uganda, Ghana, the Democratic Republic of Congo, Thailand, India, and Peru increased the threshold of tree canopy cover in their national legal definitions of forest to increase the area available for international financing of afforestation and reforestation projects associated with the Clean Development Mechanism of the United Nations Framework Convention on Climate Change (Zomer et al. 2008; Romijn et al. 2013). Consequently, areas with sparse forest cover (agroforests, small woodlots) or small, isolated natural forest fragments are no longer classified as forest and can be considered areas suitable for afforestation and reforestation (van Noordwijk et al. 2009). Few afforestation and reforestation projects have actually been carried out under the CDM mechanism due to financial, administrative, and governance issues (Thomas et al. 2010); however, leaving these newly designated “non-forest” areas susceptible to conversion to non-forest land uses. When areas in Indonesia were prioritized for the CDM Mechanism, the forestry department realized that 70 % of the land classified as forest in 1989 was not eligible because it was still defined as forest, regardless of tree cover (van Noordwijk et al. 2008).

A consequence of the minimum tree cover and area thresholds in many national and international forest definitions is that small, isolated forest patches, riparian forest strips, live fences, agroforests, and remnant trees standing within a matrix of non-forest land uses remain unrecorded (Box 1). Areas classified as “non-forests” are as important to forest definitions as are forests. More than 43 % of agricultural land globally is in agroforestry systems with >10 % tree cover (Zomer et al. 2014). In Rwanda and Brazil, forest inventories using a 0.5-ha threshold ignore substantial areas of small forest fragments, agroforests, and woodlots, leading to underestimates of actual tree cover (Nduwamungu et al. 2014; Ribeiro et al. 2009). Small patches of trees and even isolated remnant trees can hold high ecological and conservation value (Solar et al. 2015), and can play an important role in enhancing landscape connectivity, local biodiversity (Manning et al. 2006), and local livelihoods (Ndayambaje et al. 2013).

Another major policy consequence of using forest definitions based solely on forest structure is the failure to differentiate forests disturbed by logging operations from forests regrowing spontaneously on former agricultural land (Chazdon 2014). Estimates of the area of “secondary forest” in the tropics vary widely depending on whether forests recovering from logging are included in the definition (Pan et al. 2011; Achard et al. 2014). Implementation of carbon mitigation forest policies that rely heavily on the potential for carbon storage during forest regrowth will be compromised if secondary forests are not properly accounted for in national assessments. Assessing rates of spontaneous forest regrowth also provides critical information for implementing large-scale forest restoration, yet this information is lacking at regional and national scales.

Intentional or not, it is clear that the choice of forest definition has had a pervasive impact on monitoring, assessing, and interpreting forest change (Lund 2014). Clearly, forest definitions should not be used for purposes for which they are not appropriate. Definitions should instead be tailored to achieve specific policy objectives.

## Reforestation, afforestation, restoration, or rehabilitation?

Multiple coexisting forest concepts and definitions have led to a confusing array of terms for “reforests.” According to the FAO’s Forest Resource Assessment (FAO 2012), forested land area can increase through two processes: *afforestation* (planting or seeding of trees on land that was not previously forested) or *natural expansion* (expansion of forest on land previously not classified as forest). But neither of these processes is considered reforestation. As defined by the FRA, *reforestation* (re-establishment of forest through planting trees or deliberate seeding on land already classified as forest) does not increase forest area, as it occurs on lands already defined as forest. These definitions are consistent with the FRA concept of forest as land-use (Fig. 1). Forest definitions required by the Kyoto Protocol emerged from FRA forest definitions, which do not include a concept of restoration (Ma et al. 2014).

**Fig. 3:**
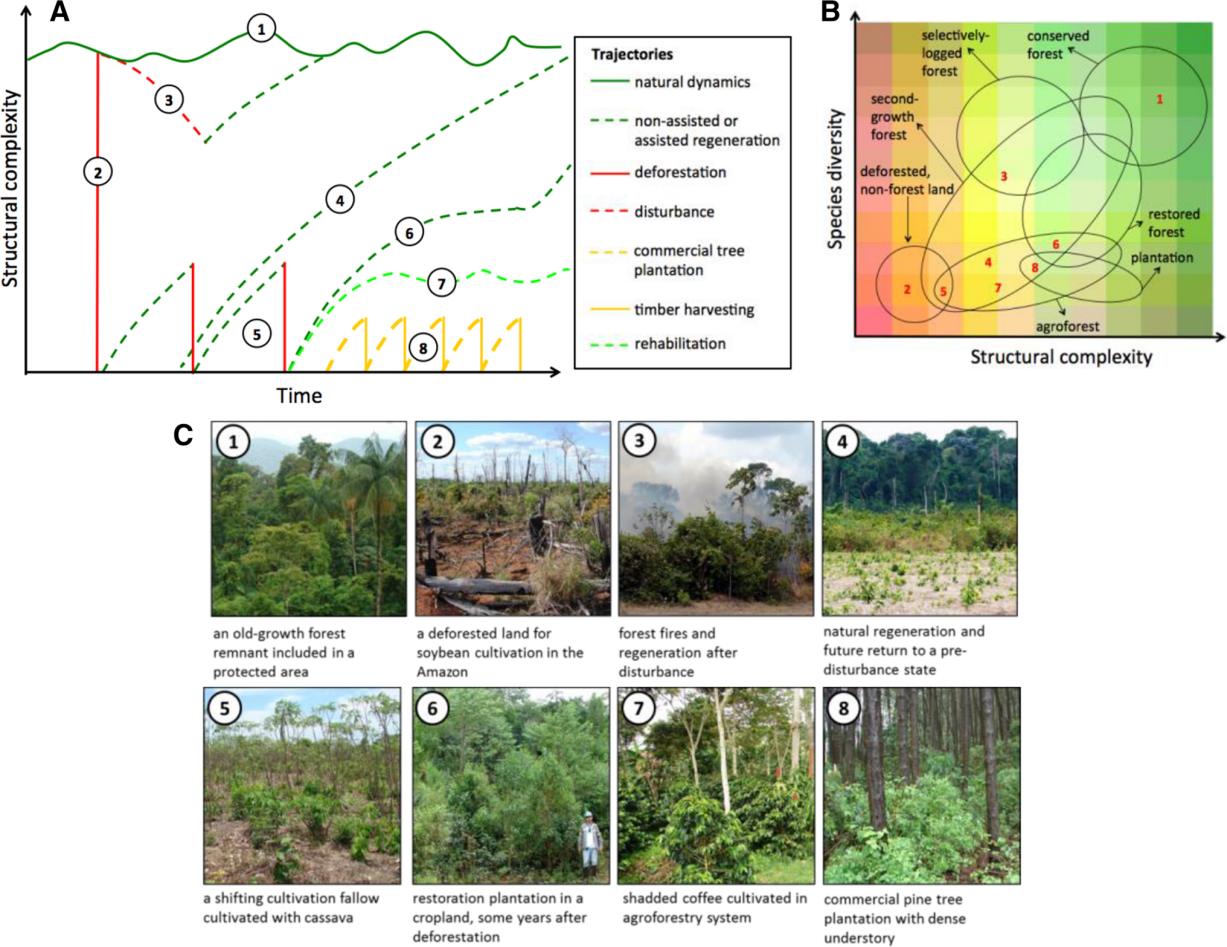
Superficially similar forest states in forests and reforests can be distinguished by their dynamic trajectories over time. **a** Forest trajectories in terms of structural complexity over time. **b** Forest states in terms of structural complexity and biological diversity. States can vary (indicated by the size of the *circles* and *ellipses*) and overlap considerably in their properties. Numbers refer to different forest states, which are illustrated in **c**. Natural dynamics (*1*) occur in conserved and remote areas. Loss of structural complexity can happen through deforestation (*2*) and disturbance (*3*). Increases of structural complexity can happen through different types of regeneration (*4*, *5*, and *6*). Agroforestry and commercial tree plantations (*7* and *8*) may have structural similarities to natural forests, but show different trajectories

A distinct set of concepts and definitions related to the reestablishment of forest cover has emerged from the field of ecology. Operational definitions for a family of “Re-” terms—restoration, recuperation, rehabilitation, etc.— originated from the wilderness preservation movement in the US, which aimed to manage ecosystems for conservation and preservation rather than extraction (Jordan and Lubick 2011). “Re-” terms are differentiated by their process and end goals, which vary in the degree to which they are true to the species composition, structure, and function of historical ecosystems (Stanturf et al. 2014). According to definitions used in many English-speaking countries, forest *restoration* emphasizes historical fidelity and recovery of native species composition (ecological integrity), whereas forest *rehabilitation* emphasizes functional aspects of recovery, and can involve non-native species (Fig. 3).

Changing views and language in the realm of reforestation and forest restoration parallel historical changes in forest management concepts and shifts in the sectors involved in research and implementation (Fig. 2). Within the forestry sector in the tropics, site-based approaches to reforestation focused on planting a few non-native tree species for timber production, rather than addressing the root causes of forest loss and degradation. In response to the widespread failure of these approaches to conserve native biodiversity, the concept of Forest Landscape Restoration (FLR) arose in 2000. FLR represented a significant departure from small-scale, stand-management approaches toward a landscape approach incorporating multiple forest functions to provide livelihoods and ecosystem services for local people (Laestadius et al. 2015). Implementing forest and landscape restoration requires a new approach to assessing forests that includes both the qualities and trajectories of forest patches in a spatial matrix of non-forest land uses, and their role in enhancing multiple management objectives at relatively large spatial scales.

The landscape approach considers forests as internally interactive landscape units in which the trajectory of a forest patch is influenced by the state of neighboring patches (Sloan et al. 2015). Restoration outcomes for a particular forest patch will heavily rely on the connectivity with other patches, while the status of these patches will also influence restoration success, for ecosystem services provisioning and biodiversity conservation alike. Landscape units not typically assessed in forest inventories, such as isolated trees, living fences, small remnant forest patches, and woodlots enhance ecological integrity and improve restoration outcomes (Botzat et al. 2015).

## Defining forests based on their origins, trajectories, and landscape context

Areas of “forest gain” described in the forest transition and forest change literature (Hansen et al. 2013) could represent commercial monoculture plantations, natural regeneration, restoration plantations, or agroforests, which vary widely in their drivers, initial states, trajectories, in the goods and services they provide to people, and in their potential to support biodiversity and to mitigate climate change (Brown and Zarin 2013; Tropek et al. 2014). Recognizing this imprecision, Global Forest Watch now uses the term “tree cover” rather than “forest cover” to monitor global gain or loss. This platform has recently begun to add coverages of oil palm plantations, pulp plantations, and other types of plantations and is now able to assess whether recent forest clearing in some countries has occurred in natural forests or in plantations (Petersen et al. 2016). There is a pressing need to further develop programs and institutions to catalyze the collection and distribution of robust information on the quality and extent of forests and reforests of all types, shapes, and sizes.

The FAO attempted to harmonize definitions of forest states and processes by merging definitions used in different settings to enable land-use classifications and assessments at larger geographic scales without insisting that all countries use exactly the same definitions (Ståhl et al. 2012). The term “other naturally regenerated forest” was applied by the FRA to refer to a wide range of forest states including selectively logged forests and degraded forests, forests regenerating following agricultural land use, forest areas recovering from fires, and planted forests with naturally regenerated trees (Putz and Redford 2010). This broad category now accounts for 65 % of total global forest cover (FAO 2015). Distinguishing and assessing forest states included within this broad category—which includes most forest states that result from forest restoration processes—is necessary to support new policies in the era of forest and landscape restoration.

To assess and monitor forest and reforests properly requires viewing them as dynamic systems. We propose that forest definitions that are sensitive to forest trajectories be integrated into monitoring forest and landscape dynamics (Fig. 3a). When viewed and defined as static states, naturally regenerating forests and forests subjected to logging can exhibit similar levels of diversity and structural complexity (Fig. 3a, b). From a dynamic perspective, however, deforestation is an abrupt removal of tree cover, whereas forest disturbance is a more gradual process that can be more rapidly reversed through natural regeneration (Fig. 3a). Following deforestation, reforestation and restoration trajectories lead to the gradual recovery of forest structural complexity, but species composition may remain distinct from that of intact forests for many decades or even centuries (Fig. 3a, b). By assessing trajectories in individual forest units, we can also assess the trajectories of entire landscapes. A landscape composed of enlarging and fusing forest units undergoing natural regeneration will have a higher potential for connectivity and biodiversity conservation than a landscape composed of isolated monoculture tree plantations, or of contracting remnant forest patches.

Definitions that are sensitive to forest dynamics provide critically needed tools for the sustainable management of diverse forest landscapes (Table 1). Indigenous forest dwellers and shifting cultivators throughout the world have developed definitions for different successional stages that reflect their management potential (Toledo et al. 2003). Harnessing local ecological knowledge to define and assess forest states offers rich possibilities. If current forest assessments are to be useful for understanding the drivers and rates of land-use change, they must incorporate definitions that include the dynamic properties of forests, their uses for local people, and their changing landscape context. Successional trajectories within a given region are highly diverse and are strongly influenced by landscape factors (Arroyo-Rodriguez et al. 2015).

Definitions are made to suit specific purposes, based on a views, concepts, and priorities. The definition of a forest is not intended to encompass the totality of what forests are (Fig. 1). Here, we present a heuristic framework to illustrate how definitions applied to specific purposes vary in the importance of adopting seven criteria: (1) value for timber; (2) value for carbon storage; (3) improving livelihoods of forest-dependent people; (4) whether forests are natural or planted; (5) whether forests are pre-existing or newly established; (6) whether forest are continuous or fragmented; and (7) whether forests are composed of native or non-native species (Table 1). We contrast four different forest management objectives, based on those shown in Fig. 1. This framework shows, for example, that distinguishing pre-existing forests from “reforests” is important for each of these views, but for very different reasons. For purposes of assessing carbon stocks and for tracking forest restoration in landscapes, additionality is an important criterion. Distinguishing between native and non-native trees in forests is not important for purposes of viewing forests solely as carbon stocks, but is very important for viewing forests as natural ecosystems (Table 1). Views and definitions of forests need to adapt to changing circumstances imposed by climate change, government policies, new scientific knowledge, or international market forces. For example, changes in the market demand for timber produced through sustainable forest management certification require modification of the criteria for defining forest as sources of certified timber. Criteria for “zero deforestation” practices will also need to be developed within this framework based on a clearly stated forest management objective. Additional criteria based on social and cultural factors can be included if these are part of forest management objectives, as is the case with timber certification schemes and certification of non-timber products. Enhancement of rural livelihoods is a fundamental principle of forest and landscape restoration. Our framework provides a flexible tool for defining and assessing forests based on multiple management criteria.

## Assessing and monitoring forest and landscape change

Monitoring rates of degradation and recovery of terrestrial ecosystems as well as tracking progress toward restoration targets demand that forests be defined in a way that is sensitive to trajectory as well as state. As forest trajectories are influenced by site history and landscape context, they are manifestations of social, economic, cultural, and political change. People that rely on the land for their lives and livelihoods tend to have deep knowledge about forest properties. In these cases, local people can significantly contribute to defining, assessing, and monitoring forests and reforests (Fig. 1). Participatory mapping, where local people describe and assess forest condition and cover, is a powerful tool for incorporating local knowledge about land cover and land-use history into local assessments of forests and tree cover and how they interact within the landscape and with other land uses. Approaches using participatory mapping as a complement to remote sensing data can be particularly valuable (Vergara-Asenjo et al. 2015).

Data-sharing technologies enable assessment of forest states and trajectories at very large scales, and represent a way forward to operationalize new forest concepts and definitions in the era of restoration. Collect Earth is an “app” within Google Earth developed by FAO as a tool for data collection through visual interpretation of satellite images (Foris 2015). This tool can be used to collect information about trees and other landscape features from multiple users familiar with regional landforms and vegetation, providing baseline data to monitor all forms of tree and forest cover within a large region using freely available, high-resolution satellite imagery. A similar tool, Geo-Wiki, generate global maps of forest cover by using a network of citizen scientists to validate land cover classifications (Schepaschenko et al. 2015). Given the widespread use of mobile phones in rural areas, mobile phone apps also have great potential to map and track multiple forest and landscape attributes at high levels of spatial and temporal resolution.

Distinguishing among different types of forests and reforests—monoculture plantations, old-growth forests, logged forests, multispecies restoration plantations, and second-growth forests—in tropical regions is critical to conserving forests and forest biodiversity (Table 1). New remote sensing tools can provide high-resolution information on canopy traits and species composition, which can and should be used to distinguish among successional stages of forests, selectively logged forests, and single-species plantations, at least at the regional scale (Fagan et al. 2015; Petersen et al. 2016). Access to this information will allow countries and international agencies to track changes in natural forest cover, and to monitor processes of restoration, rehabilitation, and afforestation within a landscape context and, consequently, make informed policy decisions. We are on the frontier of developing new ways of monitoring and assessing land cover that will provide robust indicators of the quality and origins of tree cover and enable new ways of viewing and defining forests and reforests. To see beyond the overly simplified categories of forest loss, forest degradation, and forest gain, we need to develop and apply more adapted and nuanced definitions that will deepen our understanding of the drivers and outcomes of land-use change and forest dynamics within landscapes.

Definitions should not be used for purposes beyond those for which they were intended. A young regenerating forest undergoing self-organization and increasing in structural complexity and diversity over time is not the same entity as a forest in the process of decline. Forest definitions created for timber assessment purposes are insensitive to this difference, because they are based on static forest attributes. The way forward requires that we be intentional in the way we define forests for a wider range of management objectives (Fig. 2), recognizing that definitions are designed to achieve particular goals and uses (Fig. 1; Table 1). Developing and applying definitions that enable qualitative distinctions among types and trajectories of tree cover within the context of their surrounding landscapes will allow the manifold benefits of all types of forests and reforests to be recognized, assessed, and valued.

## Electronic supplementary material

Below is the link to the electronic supplementary material.

Supplementary material 1 (PDF 233 kb)
