# Supplementary material for: When is a forest a forest? Forest concepts and definitions in the era of forest and landscape restoration
Source: Ambio. 2016 Mar 9;45(5):538–50. doi: 10.1007/s13280-016-0772-y (PMC4980317; doi:10.1007/s13280-016-0772-y)
Supplement: Supplementary file 1 — Supplementary material 1 (PDF 233 kb) [file 13280_2016_772_MOESM1_ESM.pdf]

Electronic Supplemental Information
This supplementary material has not been peer reviewed

Title: **When is a forest a forest? Forest concepts and definitions in the era of forest and landscape restoration**

Timeline of key events in forest management and conservation, indicating events that required changes in forest definitions.

**Year**	**Event**	**Forest Definitions**
1713	Publication by Hans Carl von Carlowitz of Sylvicultura Oeconomica - formulates for the first time the concept of sustainability in forestry	Von Carlowitz not only elaborated a framework for a modern forestry and wood processing sector. He created the term *Nachhaltigkeit* by referring to the concept of *nachhaltige Nutzung* ("sustainable utilization") of the forest. He provided a definition for what became in the following decades the basic understanding of forest management (translation by the author). This term has been used for describing the fundamental goals and achievements in managing forest resources.<br><br>http://e-collection.library.ethz.ch/eserv/eth:7346/eth-7346-01.pdf
1892	International Union of Forest Research Organisations (IUFRO) founded	
1940	CATIE established	
1943	Forty-four governments, meeting in Hot Springs, Virginia, (USA), commit themselves to founding a permanent organization for food and agriculture – later the Food and Agriculture Organization (FAO).	
1944	World Bank established.	
1945	United Nations Educational, Scientific and Cultural Organization (UNESCO) founded	
1945	1st FAO Conference, Québec - forestry on the agenda from the very	

	beginning	
1947	5th Commonwealth (Empire) Forestry Conference, Oxford, England	
1948	Forest resources of the world published by FAO - the first global forest inventory	Forest Lands bearing vegetative associations dominated by trees of any size, capable of producing timber or other forest products or of exerting an influence on the climate or on the water regime. Also, lands from which forests have been recently clear cut or burned, but which will be reforested in the near future. The 1947 inventory had demonstrated clearly that the distinction drawn therein between **productive and unproductive forests** was an arbitrary one.<br><br>http://www.fao.org/docrep/x5345e/x5345e03.htm#some%20definitions
1948	International Union for the Conservation of Nature (IUCN) founded as the International Union for the Protection of Nature	
1953	World forest resources – results of the inventory undertaken in 1953 published by FAO	Forest is defined as land bearing vegetative associations dominated by trees of any size. Thus the term "forest" in this inventory covers stands of very different densities. **Accessible forests** are defined as forests within reach of economic management or exploitation.
1958	Forest resources of the world 1958 published by FAO.	In the 1958 inventory forests is defined with somewhat more precision than hitherto. Thus, bamboo lands and wattle (Acacia spp.) plantations are specifically included, while savanna types with an average density of tree cover below 0.05 are excluded, as are non-forest crop lands such as rubber and cinchona.<br>This inventory defines **accessibility** in purely physical terms: "areas within reach of exploitation by existing transportation systems". Forest area alone, however, can convey a very misleading impression of the potential of the forest resource. There is obviously need for some indication, however approximate, of the **quality of the forest**.<br>The **revised definition of forests in the 1958 inventory**,

		already mentioned, to some extent sharpens the picture the inventory gives us of the forested area, by specifically excluding land with very low timber cover. In addition, a tree cover section asks countries to report the proportions of the exploited forest area in density classes.<br><br>http://www.fao.org/3/a-ad906t.pdf
1961	Worldwide Fund for Nature (WWF) founded as World Wildlife Fund in Switzerland	
	Silent Spring, by Rachel Carson, published	
1963	World forest inventory 1963 published by FAO	**Forest land**: The term forest land used in the WFI 1963 and in the FAO Production Yearbook has often been taken as synonymous with "forest and other wooded areas". The exact wording of the definitions of this term in these two publications are as follows:<br>WFI 1963: All lands bearing vegetative associations dominated by trees of any size, exploited or not, capable of producing wood or other forest products,of excerting an influence on the climate or on the water regime, or providing shelter for livestock and wildlife.<br>http://foris.fao.org/static/data/fra2010/WorldForestResources.pdf (pg 246)<br>**Forest** (stocked forest land): Forest lands bearing a tree or bamboo cover, whether productive or not.<br>http://www.fao.org/3/a-ad907t.pdf<br><br>An important new question asked in the 1963 enquiry was the area of "unstocked forest land", defined as "lands from which forests have been clear-cut or burned, but which will be reforested in the foreseeable future" and "lands not suitable for bearing tree cover by their nature, e.g. forest roads, small rivers and small water areas". It was considered desirable to know how large these areas were and to subtract them from the area to be reported on in the enquiry into density, composition and growing stock.<br>http://www.fao.org/docrep/007/ad907t/AD907T02.htm
1965	United Nations Development Programme (UNDP) established	
1970	Committee on Forestry (COFO) established by	

	FAO, the first UN meeting on matters concerning the forestry sector	
1974	World Forest Resources 1974 published by FAO	**Forest** and Other Wooded areas: Areas covered with trees and/or shrubs and not used-primarily for agriculture or other non-forestry purpose.<br>The publication of WFR was made in 1974 with a new approach. Important role of Dr Reidar Perrson to build the new approach of forest resource appraisal.<br>This is a continuation of the World Forest Inventories (WFI), which were earlier undertaken by FAO at 5-year intervals. The 1968 inventory was not finished. The information presented in the WFR report is of a new type compared to earlier versions of the WFI, but it does replace the information given in the 1963 WFI and can be thought of as a fifth unofficial version of the WFI.<br><br>http://foris.fao.org/static/data/fra2010/WorldForestResources.pdf
1975	UNEP founded	
1980	Tropical forest resources published by FAO and UNEP	FRA 1980 employed the following minimum criteria for defining forest (10 % canopy cover density, tree height of 7 m and area of 10 ha). The FAO Forest Assessment produced in the early 1980s (FAO/UNEP 1981, Lanly 1982) provides a first-order estimate of deforestation rates world-wide.
1983	International Tropical Timber Agreement (ITTA) developed, under the auspices of UNCTAD	
1986	International Conference on Forests and Trees (SILVA), Paris	
1986	International Tropical Timber Organization established, with headquarters in Yokohama, Japan	
1987	World Commission on Environment & Development (the Brundtland Commission) publishes final report, "Our Common Future," and	

	defines sustainable development	
1987	Montreal Protocol on substances that Deplete the Ozone Layer adopted (September)	
1988	Interim Report on the State of Forest Resources in the Developing Countries, published by FAO	
1988	Intergovernmental Panel on Climate Change (IPCC) established	**Forest** is a minimum area of land of 0.05 – 1.0 hectares with tree crown cover (or equivalent stocking level) of more than 10 – 30 per cent with trees with the potential to reach a minimum height of 2 – 5 metres at maturity in situ.<br><br>A forest may consist either of closed forest formations where trees of various storeys and undergrowth cover a high portion of the ground or open forest. Young natural stands and all plantations which have yet to reach a crown density of 10 – 30 per cent or tree height of 2 – 5 metres are included under forest, as are areas normally forming part of the forest area which are temporarily unstocked as a result of human intervention such as harvesting or natural causes but which are expected to revert to forest.<br>Remark: Forests are not defined for reporting under the Convention. The IPCC Guidelines encourage countries to use detailed ecosystem classifications in the calculations and in reporting broad specified categories to ensure consistency and comparability of national data across countries.<br>http://www.ipcc-nggip.iges.or.jp/public/gpglulucf/gpglulucf_files/Glossary_Acronyms_BasicInfo/Glossary.pdf
1990	Forest Resources Assessment 1990 published by FAO	Forest<br><br>Three different definitions of forest have been adopted in the analysis and presentation of survey results. (1) Closed forest only; (2) closed + open forest + 2/3 fragmented forest; (3) closed + open + fragmented + long fallow.<br>Closed: avg. ht >5 m, >40% canopy coverage; continuous;<br>Open: avg. ht > 5 m, 10-40% canopy coverage, continuous;<br>fragmented: ht > 5 m, canopy coverage >10%, Mosaic of forest and non-forest with forest fraction between 10 and 70% of total area<br>(estimated average 33%).
1992	UN Conference on	

	Environment and Development (UNCED), Rio de Janeiro, Brazil. Outcomes included the "Forest Principles", and Chapter 11 of Agenda 21 Combating Deforestation.	
1993	Convention on Biological Diversity (CBD)	The group considers the FAO definition of a forest as the basic one (FAO, 1998; FRA 2000), but acknowledge that many other useful definitions of "forest" exist in published form. The fact that "forest" has been defined in many ways is a reflection of the diversity of forests and forest ecosystems in the world and of the diversity of human approaches to forests. In this document, a forest is a land area of more than 0.5 ha, with a tree canopy cover of more than 10%, which is not primarily under agricultural or other specific non-forest land use.In the case of young forests or regions where tree growth is climatically suppressed, the trees should be capable of reaching a height of 5 m in situ, and of meeting the canopy cover requirement.
1993	Center for International Forest Research (CIFOR) established within the CGIAR	
1993	Global Environmental Fund (GEF) established by ten UN and other international agencies as an independent financial organization to address global environmental issues	
1993	UN Commission on Sustainable Development established to ensure follow-up to UNCED	
1993	Forest Stewardship Council (FSC) founded in Toronto, Canada with headquarters in Oaxaca, Mexico, now Bonn, Germany.	
1993	UN Framework Convention on Climate Change (UNFCCC) enters into force (March)	
1994	UN Framework Convention	

	on Climate Change (UNFCCC) enters into force (March)	
1995	1st FAO Ministerial Meeting on (June)	
1995	World Trade Organization (WTO) established (January)	
1995	First State of the World's Forests for the forest: research in a changing world (SOFO) published by FAO	
1997	Delegates to the UNFCC sign the Kyoto Protocol	
1997	Intergovernmental Forum on Forests (IFF) established	
1999	State of the World's Forests 1999	
2000	UN Forum on Forests (UNFF) established in succession to the IFF as a subsidiary body of ECOSOC	
	Millennium Summit, New York (September). World leaders accept the Millennium Development Goals	
2000	Global Forest Resources published by FAO Assessment, 2000 (FRA2000)	For the definition of **forest**, FAO adopted the threshold of 10 percent crown cover. Recommended in the landmark study on worldwide vegetation classifications carried out by the United Nations Educational, Scientific and Cultural Organization (UNESCO, 1973), this threshold was used for developing countries in the Forest Resources Assessments of 1980 and 1990, but **FRA 2000 was the first assessment to use it as the minimum canopy cover to describe forests in industrialized countries as well.**<br><br>To make the comparison between forest area in 1990 and 2000 possible, the 1990 national forest area figures derived by the interim 1995 assessment were revised, using the same definitions, methodologies and new inventory data used for calculating the 2000 figures. This established a new baseline for forest cover in 1990. It should be noted that updating national forest area figures from a previous

		forest resources assessment by incorporating new data is a standard feature of all FAO forest assessments.<br><br>"Forest includes natural forests and forest plantations. It is used to refer to land with a tree canopy cover of more than 10 percent and area of more than 0.5 ha. Forests are determined both by the presence of trees and the absence of other predominant land uses. The trees should be able to reach a minimum height of 5 m. Young stands that have not yet but are expected to reach a crown density of 10 percent and tree height of 5 m are included under forest, as are temporarily unstocked areas. The term includes forests used for purposes of production, protection, multiple-use or conservation (i.e. forest in national parks, nature reserves and other protected areas), as well as forest stands on agricultural lands (e.g. windbreaks and shelterbelts of trees with a width of more than 20 m), and rubberwood plantations and cork oak stands. The term specifically excludes stands of trees established primarily for agricultural production, for example fruit tree plantations. It also excludes trees planted in agroforestry systems."<br><br>http://www.fao.org/docrep/003/Y0900E/y0900e05.htm
2001	Collaborative Partnership on Forests (CPF) established, in succession to the ITFF. It consists of 14 major forest-related international organizations, institutions and convention secretariats.	
2001	Marrakesh Accords, include three main elements: A set of principles to govern LULUCF activities; Definitions for Article 3.3 activities and agreed activities under Article 3.4; and a four-tier capping system limiting the use of LULUCF activities to meet emission targets.	b) states that for land use, land-use change and forestry activities under Articles 3.3 and 3.4 of the Kyoto Protocol, the following definition of forest shall apply:<br>“Forest’ is a minimum area of land of 0.05–1.0 hectares with tree crown cover (or equivalent stocking level) of more than 10–30 per cent with trees with the potential to reach a minimum height of 2–5 m at maturity in situ. A forest may consist either of closed forest formations where trees of various storeys and undergrowth cover a high proportion of the ground or open forest. Young natural stands and all plantations which have yet to reach a crown density of 10–30 per cent or tree height of 2–5 m are included under forest, as are areas normally forming part of the forest area which are temporarily unstocked as a result of human intervention such as harvesting or natural causes

		but which are expected to revert to forest. http://unfccc.int/methods/lulucf/items/3063.php
2003	The Global Partnership on Forest Landscape Restoration was formed to bring about compromises by restoring a range of forest functions at the landscape level.	The concept of forest landscape restoration (FLR) emerged as is defined as “a planned process to regain ecological integrity and enhance human well-being in deforested or degraded landscapes.” http://en.wikipedia.org/wiki/Forest_landscape_restoration - cite_note-1<br>FLR comprises tools and procedures to integrate site-level forest restoration actions with desirable landscape-level objectives, which are decided upon via various participatory mechanisms among stakeholders. FLR is increasingly being considered in international and national strategies to address some of the major challenges of our time: food and energy security, climate change, poverty alleviation, combating desertification, biodiversity conservation and environmental conservation.<br><br>Reitbergen-McCraken, J., S. Maginnis A. Sarre, 2007. *The Forest Landscape Restoration Handbook*. Earthscan, London, 175 pp
2005	3rd FAO Ministerial Meeting on Forests	
2005	State of the World Forests 2005 published by FAO	
2005	Kyoto Protocol comes into force, includes legally binding goals for greenhouse gas emissions by developed countries, and establishes the Clean Development Mechanism (CDM) for developing economies.	For the CDM, developing countries must choose the parameter values from the ranges:<br><br>“Forest” is a minimum area of land of 0.05-1.0 hectares with tree crown cover (or equivalent stocking level) of more than 10-30 per cent with trees with the potential to reach a minimum height of 2-5 metres at maturity in situ. A forest may consist either of closed forest formations where trees of various storeys and undergrowth cover a high proportion of the ground or open forest. Young natural stands and all plantations which have yet to reach a crown density of 10-30 per cent or tree height of 2-5 meters are included under forest, as are areas normally forming part of the forest area which are temporarily unstocked as a result of human intervention such as harvesting or natural causes but which are expected to revert to forest;
2008	UN-REDD Programme launched by FAO, UNDP and UNEP	

2009	UNFCCC, Conference of Parties (COP15) and meeting of the parties to the Kyoto Protocol (CMP5) in Copenhagen. Record numbers of participants and observers but the outcome is not clear-cut – except for forestry, where REDD is established.	"Forest" is a minimum area of land of 0.05-1.0 hectares with tree crown cover (or equivalent stocking level) of more than 10-30 per cent with trees with the potential to reach a minimum height of 2-5 meters at maturity *in situ*. A forest may consist either of closed forest formations where trees of various storeys and undergrowth cover a high proportion of the ground or open forest. Young natural stands and all plantations which have yet to reach a crown density of 10-30 per cent or tree height of 2-5 meters are included under forest, as are areas normally forming part of the forest area which are temporarily unstocked as a result of human intervention such as harvesting or natural causes but which are expected to revert to forest.
2010	Global Forest Resources Assessment, 2010 (FRA2010) published by FAO	Land spanning more than 0.5 hectares with trees higher than 5 meters and a canopy cover of more than 10 percent, or trees able to reach these thresholds in situ.
2011	International Year of Forests	
2011	FAO 2011 satellite-based assessment of world forests	Global forest cover, as well as forest loss, is lower than previously estimated by the Food and Agriculture Organization (FAO), according to a new satellite-based assessment that replaces the self-reporting system previously used by the U.N. agency.<br>The survey found the world's total forest cover amounted to 3.69 billion hectares, or roughly 30 percent of global land area, in 2005. FAO's Global Forest Resources Assessment 2010 (FRA 2010) — which was based on the old system — put total forest cover at 4.06 billion hectares. FAO says its assessment does not adequately account for changes in tree cover in areas where the canopy is less than 30 percent. Forests will low canopy cover are usually degraded or occur in dry areas.<br><br>- See more at: http://news.mongabay.com/2011/1130-un_forests.html#sthash.wZqmsJem.dpuf
	UNFCC, Conference of Parties (COP17) and meeting of the parties to the Kyoto Protocol (CMP7) in Durban (South Africa)	
	The **Bonn Challenge** hosted and launched by Germany and the International Union for Conservation of Nature	Bonn Challenge is a global effort to restore 150 million hectares of the world's degraded and deforested lands by 2020. This is as a practical, action-orientated platform to facilitate the implementation of several existing

	(IUCN) in Bonn on 2 September 2011, in collaboration with the Global Partnership on Forest / Landscape Restoration.	international commitments that require restoration, including the CBD Aichi Target 15, the UNFCCC REDD+ goal and the Rio+20 land degradation target.
2012	UN Conference on Sustainable Development (Rio+20), Rio de Janeiro, Brazil	
2014	Nagoya Protocol entered into force on 12 October 2014	
2015	FRA 2015 - FAO	**FOREST -** Land spanning more than 0.5 hectares with Trees higher than 5 meters and a canopy cover of more than 10 percent, or trees able to reach these thresholds in situ.. It does not include land that is predominantly under agricultural or urban land use.<br><br>http://www.fao.org/docrep/017/ap862e/ap862e00.pdf
